# Lupus nephropathy beyond immunosuppression: Searching for nephro and cardioprotection

**DOI:** 10.3389/fneph.2023.1105676

**Published:** 2023-01-27

**Authors:** Enrique Morales, Justo Sandino, María Galindo

**Affiliations:** ^1^ Department of Nephrology, University Hospital “12 de Octubre”, Madrid, Spain; ^2^ Research Institute of University Hospital “12 de Octubre” (imas12), Madrid, Spain; ^3^ Department of Medicine, Complutense University of Madrid, Madrid, Spain; ^4^ Department of Rheumatology, University Hospital “12 de Octubre”, Madrid, Spain

**Keywords:** lupus nephritis, proteinuria, RAAS blockade, diuretics, Isglt2

## Abstract

Renal involvement in systemic lupus erythematosus (SLE) represents one of the most frequent organ manifestations, often leading to end-stage kidney disease (ESKD). Several therapies have been tested in patients with lupus nephritis (LN) to prevent further organ damage. The effectiveness of immunosuppressive therapy as a treatment for LN is abundant, supported by multiple clinical trials that have shown its efficacy in preventing the development of chronic kidney disease (CKD). In addition to immunosuppressive therapy, several traditional and recent therapies aimed at nephroprotection in patients with proteinuric chronic kidney disease are gaining importance in the setting of LN. Thus, immunosuppressive therapy should be accompanied by nephro- and cardioprotective measures to control cardiovascular risk factors and proteinuria to ensure a better renal prognosis. Despite this, the literature on these specific measures is relatively scarce, with recommendations focused on the blockade of the renin-angiotensin-aldosterone system (RAAS). This review explores the pharmacological options available for cardiovascular and renal protection outside the usual treatment schemes.

## Introduction

Systemic lupus erythematosus (SLE) is defined as a chronic autoimmune disease with alternating periods of quiescence and disease flares (1). Approximately 30% to 50% of SLE patients will develop some level of organ injury within five years of diagnosis and 50% or more within ten years of diagnosis (2).

SLE patients frequently develop lupus nephritis (LN) that can progress to end-stage renal disease (ESKD), with a risk between 10% and 30% at 15 years in patients with severe LN (classes III, IV, and V) (3).

According to the European Alliance of Associations for Rheumatology (EULAR) recommendations for disease management, the goals of SLE treatment are control of disease activity; prevention of flares and organ damage and, ultimately, prolongation of life (4). Treatment goals in patients with LN include preserving renal function and preventing ESKD; these outcomes can be assessed by renal biopsy and other clinical indicators of damage, such as estimated glomerular filtration rate (eGFR) slope and chronic kidney disease (CKD) staging (5).

In addition to immunosuppressive therapy, all classical and modern therapies aimed at nephroprotection in patients with proteinuric chronic kidney disease are gaining importance (6). Some novel treatments like the use of finerenone or sodium-glucose cotransporter-2 inhibitors (SGLT2i) in combination with the classical renin-angiotensin-aldosterone system (RAAS) blockers are promising therapies (7). Patients with LN are excluded from studies with new nephroprotective agents, leading to a gap in knowledge about the effect of these drugs on LN. For this reason, knowledge of these new therapies outside the nephrological world may significantly impact the preservation of renal function.

## Non-immunological factors for the progression of lupus nephropathy

By definition, all LN patients have CKD, but CKD progresses to ESKD in only some cases (3). Patients with LN who started life with a low number of nephrons, or have nephron loss beyond physiologic aging, have a shorter renal life span, especially in aging populations. Therefore, patients with LN and low birth weight or a previous episode of acute kidney injury are at risk for developing ESKD independent of LN disease activity. On the other hand, delay in diagnosis and adequate treatment, nonadherence to therapy, and, in the worst case, LN recurrence are factors that would lead to further nephron loss and further progression of CKD (8). On the other hand, some patients with adequate control of SLE activity may progress to ESKD after a single episode of LN, possibly due to the presence of high-risk genetic variants for CKD progression. For example, CKD risk variants of the apolipoprotein L1 (APOL1) gene are highly prevalent in afro-American individuals (9).

## Classical nephroprotective treatment

SLE patients have a higher mortality rate than the general population, with infections, cardiovascular (CV) complications, and especially renal failure as the leading causes of death (10) SLE patients present both traditional (dyslipidemia, smoking, obesity) and non-traditional (proteinuria, inflammation) CV risk factors. It is known that dyslipidemia accelerates the progressive decline of renal function in patients with CKD (11). The latest KDIGO guidelines on lipids and CKD recommend regular evaluation with a lipid profile (total cholesterol, LDL cholesterol, HDL cholesterol, triglycerides) and to initiate statin or statin/ezetimibe combination on patients aged ≥ 50 with non-dialysis-dependent CKD or a kidney transplant (12). Evidence that statins may retard CKD progression in humans is insufficient, with different outcomes reported based on the type and dose of the statin used (13). These recommendations, although strongly evidenced-supported, cannot be extrapolated to patients with CKD and SLE since data on this group of patients Is scarce.

Moreover, glucocorticoid therapy, mainly when high doses are used for a prolonged period, increases bone loss, incidence of CV events and infections, risk of malignancies, especially non-Hodgkin’s lymphoma, lung, and skin cancer (14–16). The use of hydroxychloroquine in SLE is widely extended since the benefits of this drug include lower flare rates and, particularly, lower incidence of CV events (17). Regular assessment of these risk factors and opportune treatment is crucial to avoid CV complications in patients with LN; some tools such as the Framingham risk score and the recalibrated SCORE prediction model are two widely used tools that provide estimates of the risk of developing CV events (18); however, these scales did not include lupus nephritis or steroid use into their models. Some studies (19, 20) show that the QRISK3 calculator could be more accurate in predicting cardiovascular events in SLE patients, recommending measures during the initial stages of disease.

Pregnancy in patients with LN is associated with increased maternal complications compared to the healthy population (21) Prompt contraceptive and pregnancy counseling are strongly advised in SLE patients of childbearing age. Furthermore, low-dose aspirin has been shown to have a protective effect in patients with SLE during pregnancy and reduce the incidence of CV events in patients with associated antiphospholipid syndrome (APS) (4).

The amount of proteinuria is a decisive factor in the prognosis of patients with LN. The goal is to achieve proteinuria <0.7 g/24h (or protein/creatinine ratio <0.7 g/g) within the first year of treatment and complete remission (proteinuria <0.5 g/24h or PCR <0.5 g/g) during the clinical course (4). Treating LN and proteinuria with RAAS blockade at maximum tolerated doses is recommended, complemented with a sodium-poor diet to optimize its effect (10). Moreover, the 2019 EULAR-ERA EDTA recommendations for the management of LN suggest that RAAS blockade is recommended (in non-pregnant patients) due to its anti-proteinuric and antihypertensive effects; with no specific recommendation for any particular drug, either angiotensin-converting enzyme inhibitors (ACEIs) or angiotensin II receptor blockers (ARBs) (4, 6) In fact, recent studies suggest that early RAAS blockade introduction is associated with an earlier rate of glucocorticoid discontinuation (22). Kanda and collaborators demonstrated that proteinuria decreased after ARB treatment in 83% of the patients with LN (PCR decrease from 2530 to 459 g/g [P = 0.03]). In addition, serum albumin and cholesterol levels were significantly improved. Systolic blood pressure significantly decreased, with no symptoms of hypotension. The anti-proteinuric effect of ARB did not correlate with blood pressure reduction. Interestingly, higher total complement activity levels before ARB treatment were associated with a more significant reduction of proteinuria. These findings show that adding ARB would be a safe and effective treatment for LN with persistent proteinuria despite corticosteroids and immunosuppressive treatment (23).

The evidence on RAAS inhibition in recent immunosuppression-based trials shows that treatment in terms of nephroprotection is usually poorly optimized before induction therapy is initiated; both BLISS-LN and AURORA 1 trials, essential and recent studies on the use of belimumab and voclosporin on patients with LN, show that not all patients had RAAS blockade prior to randomization (66% of patients on the belimumab arm in BLISS-LN) (24, 25).

Additionally, we must remember that diuretics can be an essential tool to mitigate proteinuria, especially if combined with RAAS blockade. The use of thiazide diuretics can be particularly beneficial in patients on a high-sodium diet or as an add-on therapy in cases with significant residual albuminuria, with similar effects for thiazide-like agents. The role of loop diuretics as anti-proteinuric agents is not entirely clear, but their mechanism to reduce proteinuria is probably related to RAAS blockade and intra-glomerular pressure reduction. Data on other diuretics like amiloride, acetazolamide, and triamterene as anti-proteinuric agents are partially clear (26).

The role of mineralocorticoid receptor (MR) antagonist (MRA) in treating proteinuric kidney disease is widely known and has been described extensively (27–29). Most landmark trials did not include patients with LN since active immunosuppressive treatment is a common exclusion criterion.

Evidence indicates that the use of spironolactone on top of ACEIs and ARBs induces a proteinuria reduction of approximately 30–40% (30, 31), with an initial decline of (eGFR) with subsequent stabilization (29). Hyperkalemia is a frequently feared side effect of MRA treatment, especially when combined with RAAS blockade. However, evidence supports that discontinuation of MRA after an episode of hyperkalemia is associated with higher mortality compared to continuing MRA therapy, an option to be considered, especially since the new potassium binders became available (32).

Non-steroidal anti-inflammatory drugs (NSAIDs) are a cornerstone therapy for several symptoms of SLE, although it must never be overlooked that NSAIDs are nephrotoxic. In addition, LN is a risk factor for NSAID-induced acute kidney injury, and NSAID-induced hepatotoxicity is increased in SLE patients (33). This makes it crucial to minimize or avoid using NSAIDs and other nephrotoxic agents in patients with SLE and established CKD, with special care on dose adjustment for several agents commonly used for nephro-protection. (**Table 1**)

**Table 1:** Commonly used drugs for nephro and cardioprotection in patients with lupus nephritis.

Drugs commonly used in patients with SLE	Special considerations on patients with CKD
**NSAIDs**	Avoid or minimize its use
**Antimalarial (Hydroxychloroquine)**	Dose adjustment might be necessary to avoid retinopathy.
**RAAS blockers** *ACEi* *ARB* *MRA*	Might induce AKI High risk of hyperkalemia and hypotension Special care in patients with known renal artery stenosis Hepatic toxicity
**Diuretics**	Might induce AKI Electrolytic disorders (hypokalemia, hypomagnesemia) High risk of hypotension
**Metformin**	Avoid when GFR is < 30 ml/min Risk of hypoglycemia and lactic acidosis
**SGLT-2i**	Avoid when GFR is < 25 ml/min. Risk of hypotension and diabetic ketoacidosis Risk of urinary tract and skin infections.
**GLP-1RA**	No dose adjustment is required but not recommended in patients on dialysis.

SLE, Systemic lupus erythematosus; CKD, chronic kidney disease; NSAIDs, Non-steroidal anti-inflammatory drugs; RAAS, renin-angiotensin-aldosterone system; ACEi, Angiotensin-converting enzyme (ACE) inhibitors; ARB, angiotensin II receptor blockers; MRA, mineralocorticoid receptor antagonist; AKI, acute kidney injury; GFR, glomerular filtrate rate; SGLT-2i, Sodium-glucose cotransporter-2 inhibitors; GLP-1RA, Glucagon-like peptide-1 receptor agonists.

## Future perspectives in terms of nephro-protection in lupus nephritis

### Finerenone

Novel MRA, like finerenone, are known to block MR-mediated sodium reabsorption and MR overactivation with additionally demonstrated anti-inflammatory and anti-fibrotic effects (34). Recent data from the FIDELITY pooled analysis of the FIDELIO and FIGARO trials found reductions in CV events and kidney failure outcomes with finerenone in type-2 diabetic patients with CKD. In fact, in the FIDELIO-DKD trial, finerenone was associated with a more significant reduction in albuminuria from baseline to month 4, which was maintained subsequently (35). These beneficial effects on reducing proteinuria and cardiovascular events may be extrapolated to patients with LN. However, there are no data on the use of finerenone in this specific population, so proper clinical trials are needed to establish accurate recommendations.

### SGLT2i

Sodium-glucose cotransporter-2 inhibitors (SGLT2i) have recently been demonstrated to exert profound cardio and nephroprotection in large cardiovascular outcome trials (36). In patients with heart failure, independent of whether they have or not type 2 diabetes, SGLT2i reduces the progression of CKD and improves CV outcomes (36). Since many aetiologies of non-diabetic nephropathy are characterized by intraglomerular hypertension (37), we hypothesize that SGLT2i acutely decreases GFR and proteinuria in patients without diabetes at risk of progressive kidney function loss *via* a glucose-independent hemodynamic mechanism. Furthermore, distinct complications of SLE are also amenable to the therapeutic potential of SGLT2i, such as the increased occurrence of pulmonary hypertension, metabolic syndrome, and increased blood pressure. Patients with LN were excluded from such studies due to the potential necessity of acute immunosuppression (36).

Recently, we have published a pilot study in patients with LN on stable immunosuppressive treatment and non-immunosuppressive treatment with RAASi to whom a 10-mg dose of empagliflozin was added, obtaining a 50% reduction in residual proteinuria with minimal changes in eFGR and with few side effects (38).

### GLP-1RA

Glucagon-like peptide one receptor agonists (GLP-1RA) is a class B G protein-coupled receptor expressed in the pancreas and central nervous system but is also present in other organs such as the gut, kidneys, lungs, liver, heart, muscle, and peripheral nerves. GLP-1 increases insulin secretion in response to nutrients and suppresses glucagon secretion from pancreatic islet cells, reducing postprandial glucose levels (39).

Some studies suggest that GLP-1 RAs may benefit kidney outcomes, especially in improving albuminuria (40). The REWIND trial studied the effect of dulaglutide vs. placebo on the type 2 diabetic population. Only 7.9% of patients had microalbuminuria at baseline, and after a median follow-up of 5.4 years, dulaglutide proved to have a protective effect (HR 0.77, 95% CI 0.77-0.93; p=0.0004) for the appearance of new-onset albuminuria (41). Furthermore, in the AWARD-7 study, dulaglutide was shown to halt kidney disease progression and prevent the worsening of albuminuria in the diabetic population with CKD (42). As mentioned, proper clinical trials involving patients with LN are needed to establish accurate recommendations on using GLP-1 RAs as nephroprotective agents.

### Others

Endothelin receptor antagonists (ERAs) such as atrasentan and eprosartan have been associated with renal protection when combined with RAAS blockade (43). A low dose (0.75 mg/d) of atrasentan has been shown to lower albuminuria by 36% without significant side effects in subjects with type 2 diabetes with CKD who were already treated with the maximal tolerated dose of ACE inhibitors or ARBs (44). The mechanism of their renoprotective effect is believed to be based on their vascular effects, which cause glomerular vasodilation, lowering the tubular load of albumin. Moreover, endothelin has been associated with renal inflammation; endothelin receptor blockade controls renal inflammation by moderating the inflammatory effects of albuminuria reabsorption. Furthermore, the endothelin system has been implicated in the deposition of collagen and fibrosis (45). This can be of particular interest to patients with LN.

## Conclusions

**Figure 1:**
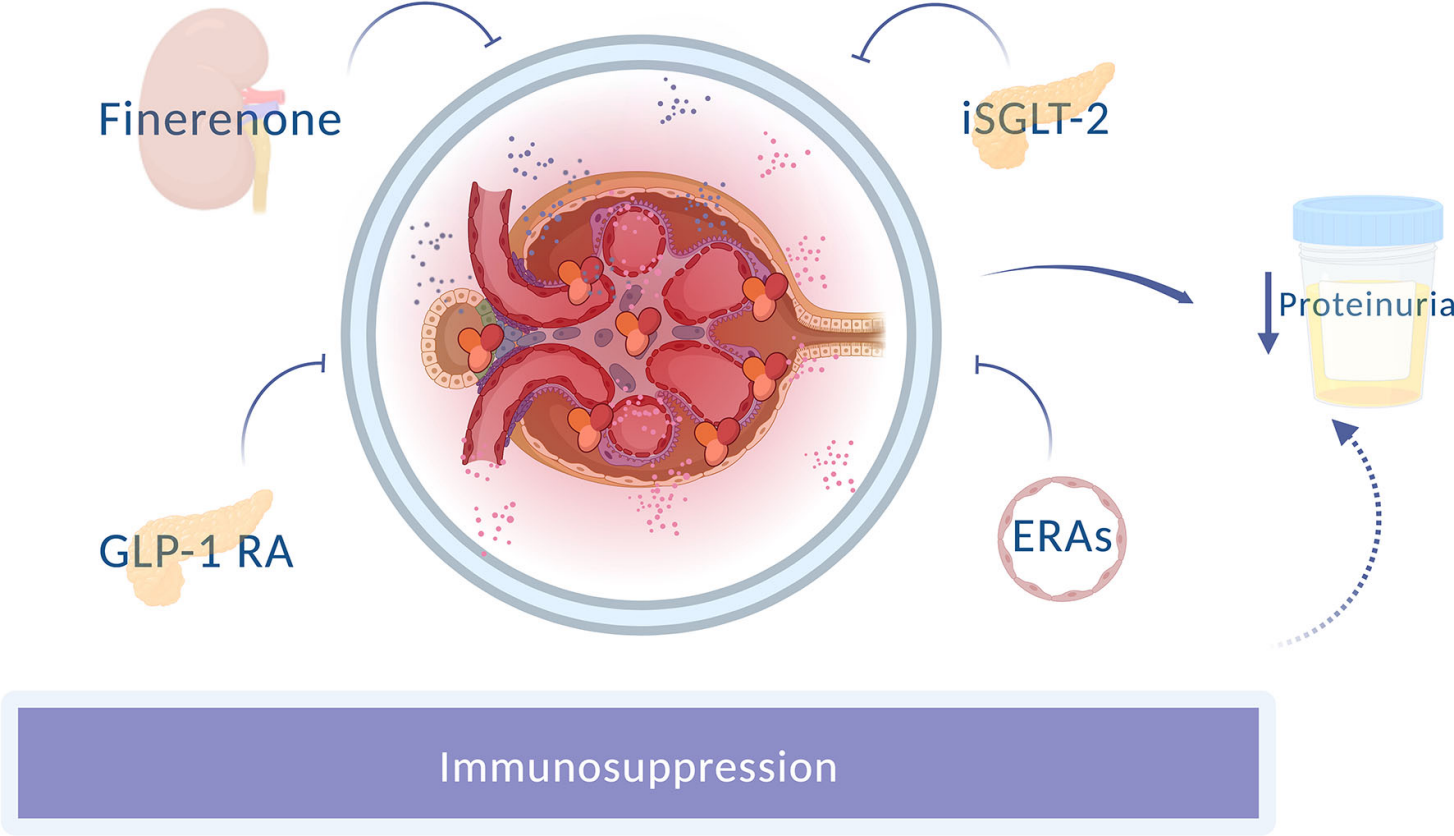
Novel nephron-protective strategies in lupus nephritis. Different drugs with an anti-proteinuric effect improve renal prognosis, apart from the usual immunosuppression. GLP-1RA, Glucagon-like peptide 1 receptor agonists; SGLT-2i, Sodium-glucose cotransporter-2 inhibitor; ERAs, Endothelin receptor antagonists.

Nephroprotection is a cornerstone in preserving renal damage in patients with lupus nephritis (**Figure 1**). In the search for comprehensive treatment, knowledge of the different therapeutic alternatives aiming at a double nephro and cardioprotection effect is crucial to avoid excessive immunosuppression and to achieve personalized and precise medical practice in our patients with lupus nephritis.

## Author contributions

All authors listed have made a substantial, direct, and intellectual contribution to the work and approved it for publication.
